# The Use of Beta-Human Chorionic Gonadotropin (β-hCG) Levels as a Predictor of Successful Medical Management of Ectopic Pregnancy

**DOI:** 10.7759/cureus.22194

**Published:** 2022-02-14

**Authors:** Ahmed S Keshta, Dalal Alarabi, Rafiea Jeddy, Maryam M Almusalam, Noor Albastaki, Aysha Alsadoon, Warda Mustafa, Haya Albuainain, Nayla Bushaqer, Nawal M Dayoub

**Affiliations:** 1 Obstetrics and Gynaecology, Royal College of Surgeons in Ireland - Bahrain, Busaiteen, BHR; 2 Obstetrics and Gynaecology, Bahrain Defense Force (BDF) Hospital, Riffa, BHR; 3 Obstetrics and Gynaecology, Assisted Reproduction and Gynaecology Centre, London, GBR

**Keywords:** treatment outcome, hcg, human chorionic gonadotropin, methotrexate, ectopic pregnancy

## Abstract

Objectives

The early diagnosis of ectopic pregnancy is essential in determining the appropriate therapeutic approach. This study demonstrates the important factors considered in the prediction of a successful medical treatment, which will, in turn, improve the quality of patient counseling and guidance prior to the initiation of the treatment.

Methods

This was a retrospective cohort study of 58 ectopic pregnancies that were treated medically with methotrexate in Bahrain Defense Force (BDF) Hospital from January 2016 to January 2021. All patients that were offered medical treatment of ectopic pregnancy and completed the follow-up were included in the study. StatsDirect software was used to analyze the baseline characteristics of the successful and failed medical treatment of ectopic groups. Simple linear regression was used to correlate initial beta-human chorionic gonadotropin (β-hCG) levels and the drop of β-hCG levels after one week of medical treatment.

Results

Patients were divided into two outcomes: the primary outcome represented in the successful treatment group, 68.9% (40/58), and the secondary outcome represented in the unsuccessful treatment group 31% (18/58). The mean β-hCG level in the successful group was significantly lower than that of the unsuccessful treatment group (1403.6±1421 IU/L versus 2845.1±1705 IU/L, p=0.001). There were no differences between the two groups with regards to the size of the adnexal mass, presence of gestational sac, or size of the gestational sac. The cut-off value of the initial β-hCG level for successful medical treatment was 2,141 IU/L, with 72% sensitivity, 75% specificity, and receiver operator curve (ROC) of 0.76 [95% confidence interval (CI) = 0.63 to 0.89)]. The cut-off value of β-hCG fell between day four and day seven and was 37.2%, with 78% sensitivity, 68% specificity, and a ROC curve of 0.72 (95% CI = 0.55 to 0.89).

Conclusion

This study found that low initial β-hCG levels can be used to predict successful methotrexate treatment of ectopic pregnancy. In this cohort of patients, the cut-off level of initial β-hCG for successful treatment was 2141 IU/L.

## Introduction

Ectopic pregnancy (EP) is potentially a life-threatening emergency that requires immediate intervention to prevent the associated risks of maternal mortality and morbidity. According to the Centers for Disease Control and Prevention (CDC), ectopic pregnancy represents 9% of all deaths related to pregnancy [1,2]. The increased incidence of ectopic pregnancy is strongly associated with previous ectopic pregnancy and previous tubal surgery. Other factors which are of slightly increased risk of occurrence of EP include pelvic inflammatory disease (PID), number of sexual partners, previous spontaneous or medical abortion, and a diagnosis of infertility [3].

The treatment options for ectopic pregnancy include medical, surgical, or expectant management. Strict case selection is essential for the initiation of medical treatment, methotrexate (MTX), that requires hemodynamic stability and an unruptured tubal pregnancy. The absolute contraindications of methotrexate therapy are those where methotrexate may cause harm such as thrombocytopenia, renal and liver disease, immunodeficiency, active pulmonary disease, peptic ulcer disease, and breastfeeding. In addition, beta-human chorionic gonadotropin (β-hCG) greater than 5,000 IU/L, the presence of fetal cardiac activity and the refusal to accept a blood transfusion are relative contraindications to methotrexate. According to the American College of Obstetricians and Gynecologists (ACOG), the three most commonly used protocols for methotrexate therapy are single dose, two doses, and multiple doses protocols. A single-dose regimen is administered intramuscularly (IM) with a standard dose of 50mg/m^2^ of MTX on day one with post-treatment follow-ups on days four and seven. For the two-dose regimen, IM administration of MTX takes place on days one and four with additional doses on days seven and eleven if the fall in the β-hCG level was less than 15%. Finally, the multiple-dose protocol requires four doses of 1 mg/kg body weight on days one, three, five, seven alternating with 0.1 mg/kg Leucovorin on days two, four, six, eight followed by a β-hCG level [4].

To date, the advantages of the different protocols are unclear due to a lack of comparative studies, but it is suggested that the two-dose protocol is more successful than the single-dose protocol according to a recently published meta-analysis [5]. Several studies have proved the safety and efficacy of methotrexate after being tested on groups of women worldwide. In contrast to the surgical intervention, medical treatment results in fallopian tube sparing and a reduced cost compared to surgery [6-8]. When administering methotrexate treatment, it is important to consider the suitability of that approach and the risk of rupture ectopic should be closely monitored. The prediction of either the rupture of the ectopic pregnancy or the success of the treatment, is reliably dependent on the change in the β-hCG levels [9].

The available literature on the medical management of ectopic pregnancy is scant and limited to a few parameters related to the management protocols. A PubMed search using the term “ectopic pregnancy” provides results that are almost ten times higher than using the terms “ectopic pregnancy” AND “methotrexate”. This gap refers to the lack of studies discussing the management of ectopic pregnancy comprehensively. In this study we evaluate the medical management of ectopic pregnancy. This includes the evaluation of patient characteristics presenting with different β-hCG measurements, signs and symptoms and mass size between successful and unsuccessful medical treatment. Additionally, we evaluated different β-hCG interval-measurements to enable cut-off values for the prediction of methotrexate treatment success.

## Materials and methods

This was a retrospective cohort study of 67 women with ectopic pregnancies that had been treated medically with methotrexate (MTX) in the department of Obstetrics and Gynecology BDF Hospital from January 2016 to January 2021. Fifty-eight women were identified as being eligible for inclusion in the study, while nine were excluded either due to the withdrawal of medical treatment, a history of renal disease, shifting to surgical intervention, missing data from the BDF hospital electronic medical records, and the loss of follow-up after the completion of the treatment course. The diagnosis of ectopic pregnancy was made by assessment of clinical parameters represented in lower abdominal pain, vaginal bleeding, adnexal mass palpation, rebound tenderness, transvaginal ultrasound findings of the size and location of the gestational sac, and β-hCG measurement prior to MTX initiation. All patients consented to receive MTX for ectopic pregnancy. This study was approved by the Research Ethics Committee of BDF hospital. The inclusion criteria of the BDF Hospital for treating ectopic pregnancy medically were as follows: patients who were hemodynamically stable with β-hCG levels below 3000 mIU/ml, adnexal mass less than 3.5 cm, little or no hemoperitoneum, and no fetal cardiac activity detected on ultrasound. Baseline investigations such as full blood count, β-hCG, renal and liver function tests were performed for all the patients on day one and a single dose of 50 mg/m^2^ methotrexate was administered. Follow-up assessments were then performed on days one, four, and seven then weekly until the ectopic pregnancy completely resolved. Follow-up assessments included blood β-hCG measurements, ultrasound imaging, assessment of general health and symptoms such as abdominal pain, and evaluation for side effects of methotrexate such as liver and renal deranged function tests. Patients were given another dose of MTX if the decrease in β-hCG levels between day four and day seven was less than 15%. Methotrexate success was described by Levin et al. as the complete negative β-hCG level after MTX injection and the failure with the need to perform surgery, a repeated MTX injection was not considered as failure [10].

Data were analyzed using StatsDirect statistical package version 3.3.5 (Merseyside, UK). A two-sided unpaired t-test was used to compare normally distributed continuous variables between the two groups. A two-sided Mann-Whitney test was used to compare non-normally parametric variables between the groups. Chi-square in crosstabs was used to compare percentages in each group. Fisher- Freeman-Halton exact in crosstabs was used to compare percentages between the groups when one out of four cells have an expectation of less than five. Two-sided simple linear regression analysis was used to assess the correlation between initial β-hCG levels and the drop in hCG level between day one to day seven after medical treatment. The area under the receiver operator curves (ROC) was used to evaluate the predictive value of initial β-hCG levels and the successful medical treatment. The ROC was used as well to evaluate the predictive value of the β-hCG fall and successful treatment. ROC was presented as an optimum cut-off point and 95% confidence interval (CI) with sensitivity and specificity. P-values of less than 0.05 were considered statistically significant.

## Results

A total of 67 women with ectopic pregnancies were treated with MTX; however, nine patients were excluded from the study due to lack of information in their electronic records (n=6), or medical treatment was withdrawn as the surgical intervention was necessary (n=3). The patients were then divided into two groups based on the final treatment outcome: 68.9% (40/58) were successfully treated with methotrexate, while 31% (18/58) had a failed treatment. Thirty-Nine patients (67.2%) were Bahraini and 19 (32.7%) were non- Bahraini. Out of those that were non- Bahraini, 10 (55.6) had unsuccessful MTX treatment compared to eight (44%) of the Bahrainis (Table 1, p=0.01). The baseline characteristics for the successful and unsuccessful treatment groups are shown in Table 1. The mean number of previous ectopic pregnancies was higher in the unsuccessful treatment group, but that did not reach statistical significance (p=0.06). There were no differences in the other baseline characteristics of the two groups (Table 1).

**Table 1:** Baseline Characteristics of Women with Ectopic Pregnancies a: Unpaired t-test, b: Mann-Whitney U test, c: Chi-square, d: Fisher-Freeman-Halton exact, sd: standard deviation, -ve: negative, +ve: positive, IVF: In vitro fertilization

Patient Characteristics	Successful N=40	Unsuccessful N=18	P value
Maternal age mean ± SD	31.3±7.4	34.3±7.3	0.15^a^
Nationality			0.01^c^
Bahraini	31 (77.5%)	8 (44.4%)	
Non-Bahraini	9 (22.5%)	10 (55.6%)	
Gravida mean ± SD	3.7±3.2	4.4±4.1	0.45^a^
Parity median (range)	2 (0-9)	1 (0-5)	0.73^b^
Miscarriage mean± SD	0.73±1.9	1.39±3.2	0.42^a^
Ectopic mean ± SD	0.23±0.53	0.72±1	0.06^a^
Blood group			0.46^d^
A	13 (32.5%)	4 (22.2%)	
AB	1 (2.5%)	1 (5.6%)	
B	11 (27.5%)	3 (16.7%)	
O	15 (37.5%)	10 (55.5%)	
RH blood type			0.55^d^
-ve	3 (7.5%)	0 (0%)	
+ve	37 (92.5%)	18 (100%)	
IVF	8 (19.5%)	5 (27.8%)	0.51^d^

The mean β-hCG concentration in the successful group was lower than the mean β-hCG concentration in the unsuccessful group 1403.6±1421 IU/L versus 2845.1±1705 IU/L (Table 2, p=0.001). There were no differences between the two groups in other ectopic pregnancy characteristics such as presentation with abdominal pain, bleeding, tenderness, adnexal mass, presence of gestational sac, and size of the gestational sac (Table 2).

**Table 2:** Characteristics of Ectopic Pregnancy of Women treated Medically with Methotrexate a: Unpaired t-test, b:  Chi-square, c: Fisher-Freeman-Halton exact, sd: standard deviation

	Successful N=40	Unsuccessful N=18	P value
β-hCG level mean± SD (IU/L)	1403.6±1421	2845.1±1705	0.001^a^
Lower abdominal pain	29 (72.5%)	13 (72.2%)	>0.99^c^
Vaginal bleeding	33 (82.5%)	15 (83.3%)	>0.99^c^
Rebound tenderness	16 (40%)	8 (44.4%)	0.75^b^
Palpation of adnexal mass	13 (32.5%)	6 (33.3%)	0.95^b^
Transvaginal ultrasound finding of gestation sac on scan	6 (15%)	4 (22.2%)	0.48^c^
Size of gestational sac mean± SD (cm)	0.13±0.58	0.14±0.53	0.96^a^

The mean courses of MTX administered, and the doses in the successful and unsuccessful treatment groups are shown in Table 3. The changes in β-hCG level between day one and seven (β-hCG_d1-d7_), between day four and seven (β-hCG_d4-d7_), and between day seven and 14 (β-hCG_d7-d14_) were calculated. Negative numbers indicate that the β-hCG level decreased (Table 3). Patients in the successful treatment group had a greater decrease in the β-hCG level. An unpaired t-test showed statistically significant differences between the two groups in the fall of β-hCG level between day one and seven (Table 3, p=0.003) and between day four and seven (Table 3, p=0.01). After day seven, the change in β-hCG level between the two groups did not differ. The mean time of resolution was the same in the two groups.

**Table 3:** Relationship Between β-hCG Level and Treatment Outcomes a: Unpaired t-test, β-hCG: beta-human chorionic gonadotropin

	Successful 40	Unsuccessful 18	P value
N (number) of methotrexate courses mean± SD	1.65±0.8	1.72±0.8	0.76^a^
Methotrexate dose mean± SD (mg/m^2^)	87±16.9	92.8±12	0.197^a^
β-hCG _d1-d7_ mean± SD (IU/L)	-50.1%±37.4	-10.9%±49.4	0.003^a^
β-hCG​​​​​​​ _d4-d7_ mean± SD (IU/L)	-47.5%±23.3	-28.3%±23.6	0.01^a^
β-hCG​​​​​​​ _d7-d14 _mean± SD (IU/L)	-68.6%±38.8	-56.3%±25.8	0.31^a^
Time to resolution (days) mean± SD (IU/L)	19.6±9.7	19±3.4	0.799^a^

**Figure 1:**
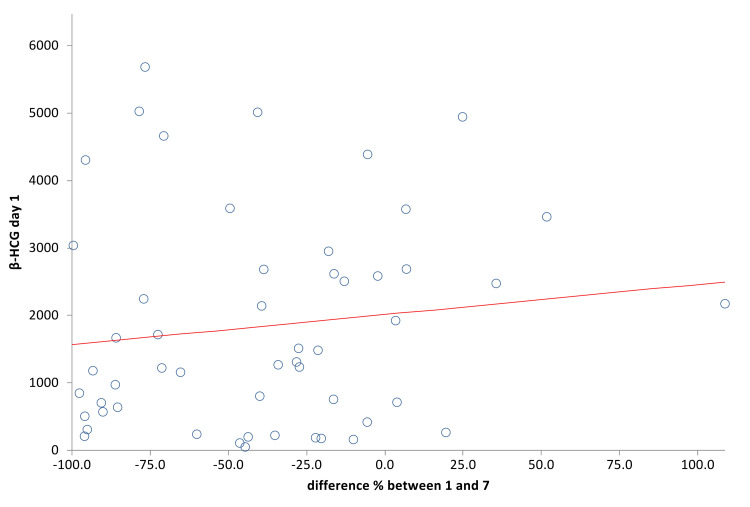
Simple Linear Regression of β-hCG Day 1 vs Difference of β-hCG From Day 1 to Day 7

A simple linear regression was plotted to assess the correlation between the initial β-hCG (day one) and the drop in β-hCG from day one to day seven (Figure 1). There was no significant relationship between initial β-hCG and the change from day one to day seven (p=0.36). Correlation coefficient (r) = -0.039

**Figure 2:**
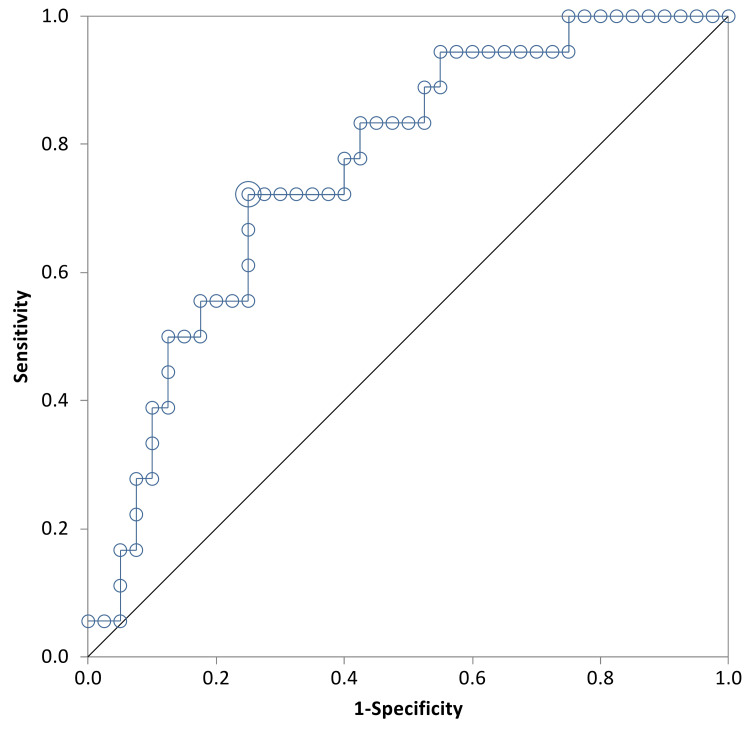
ROC Plot of Initial β-hCG Level And Successful Treatment β-hCG day (+ve), β-hCG day 1 (-ve), +ve: positive, -ve: negative, ROC: receiver operator curve

This study showed the initial β-hCG level to be a better predictor of successful medical treatment by MTX than the drop of β-hCG levels between day one to day seven and between day four to day seven. The area under the ROC curve for initial β-hCG level and successful medical treatment was 0.76 (95% CI = 0.63 to 0.89) (Figure 2). The optimum cut-off value of the initial β-hCG level to predict successful treatment was 2,141 IU/L, this cut-off point provided a sensitivity of 72% and specificity of 75%. The positive predictive value was 56.52% and the negative predictive value was 85.71%.

**Figure 3:**
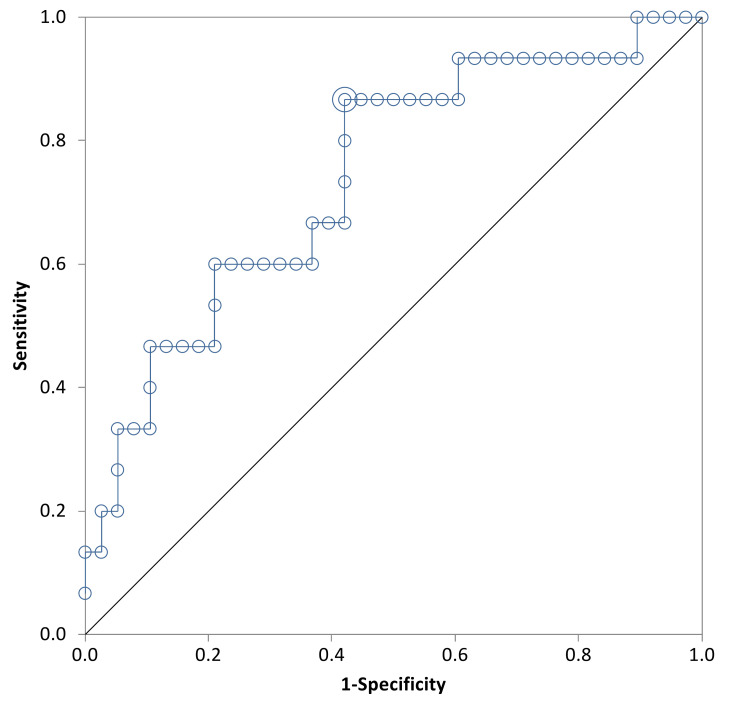
ROC Plot of β-hCG Change Between Day 1 and 7 And Successful Treatment Difference % between 1 and 7 (+ve), difference % between 1 and 7 (-ve), +ve: positive, -ve: negative, ROC: receiver operator curve

**Figure 4:**
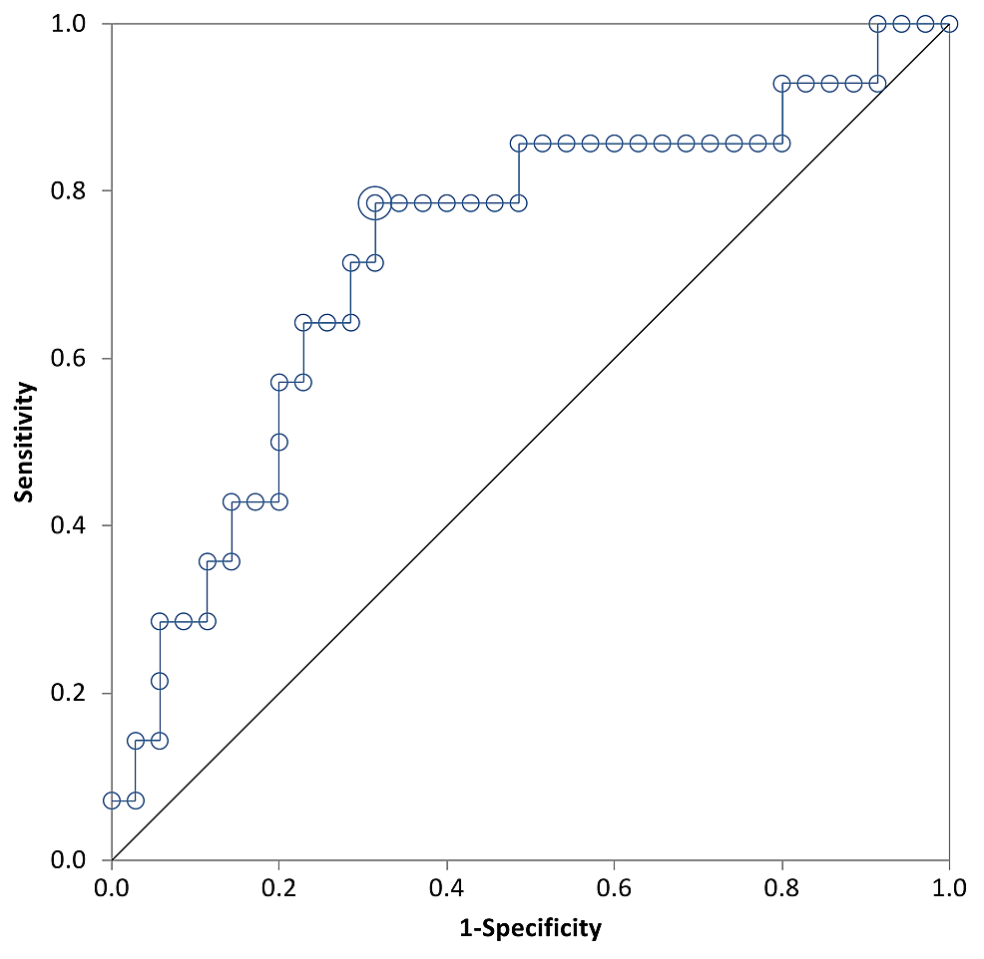
ROC Plot of the Difference of β-hCG From Day 4 to Day 7 And Successful Treatment Difference between day 4 and 7 (%)(+ve), Difference between day 4 and 7 (%)(-ve), +ve: positive, -ve: negative

The area under the ROC curve for the change of β-hCG level between day one and day seven was 0.74 (95% CI = 0.58 to 0.89) (Figure 3). The optimum cut-off value of the change in β-hCG between day one and day seven to predict successful treatment was -40.6%, this cut-off point provided a sensitivity of 86% and a specificity of 57%. The positive predictive value was 44.83% and the negative predictive value was 91.67%. The area under the ROC curve for the change of β-hCG level between day four and day seven was 0.72 (95% CI = 0.55to 0.89) (Figure 4). The optimum cut-off value for the change in β-hCG between day four and day seven was -37.2%, this cut-off point provided a sensitivity of 78% and a specificity of 68%. The positive predictive value was 50% and the negative predictive value was 88.89%.

## Discussion

Ectopic pregnancy has a prevalence of around 1-2 % worldwide with more than 90% of the cases in the fallopian tubes [11]. It is still one of the most common causes of maternal mortality and morbidity with about 50% of patients being initially asymptomatic, an early diagnosis and intervention are very crucial. Transvaginal ultrasound and serial serum β-hCG are optimal for early diagnosis and effective treatment. Different studies have looked into various factors determining the predictors of successful treatment [12,13]. This includes patients symptoms, baseline initial β-hCG levels and day four β-hCG levels, the size of the mass, a history of previous ectopic pregnancy and the route of methotrexate. In a more extensive study of 1327 patients, the success rate was up to 89% with administering multiple doses of methotrexate rather than a single dose regime [14].

In a series of studies, the initial β-hCG level alone is considered as a portal to offer medical treatment with MTX. These studies demonstrate a success rate of 65% to 95% with a mean success rate of 82% and fertility rate of successful pregnancy after treatment as 67% to 80.7% which is comparable to the surgical treatment arm [6]. In our series the principal indicator of success was the initial β-hCG, then the decline in β-hCG between day one to day seven and between day four and seven. Our study had a total 67 patients initially. However, nine were excluded as they did not fill the inclusion criteria. Out of the 58 patients considered eligible for the inclusion criteria, 40 patients (68.9%) were successfully treated with methotrexate whereas 18 patients(31 %) had failed treatment.

The two most prominent features of the patients whose treatment was not successful in our series were an initial baseline of higher β-hCG levels and a previous history of ectopic pregnancy. In the successful group, the mean β-hcg was lower. The cut off value of β-hCG, 2141 IU/L, served as a good early measure of treatment success with a specificity of 75% and a sensitivity of 72%. In another retrospective study of ectopic pregnancies a cut off of initial β-hCG greater than 3000 IU/L showed an 85.3% success rate [15]. Although our sensitivity and specificity cut offs are not high enough to completely reassure patients prior to embarking on MTX treatment, it can be used for patients counselling and guidance of the treatment with full informed consent. In addition, results published by Zhang et al. at Shaanxi Provincial People’s Hospital have shown that the overall success rate of MTX therapy for ectopic pregnancy is 69.75% with a total number of 238 patients in a one-year retrospective study from January 2017 to December 2017. Interestingly, the mean β-hCG level in the successful group was 2538 IU/L which is comparable with our series [6]. Furthermore, the mean number of previous ectopic pregnancies was higher in the unsuccessful group, in our series but did not reach statistical significance (Table 1, p=0.06).

In contrast, Natale et al. reported that there was no significant difference in the initial β-hCG in the outcomes of successful versus unsuccessful cases [16]. Consistently with most studies, variables like parity, age of the patient and previous miscarriages did not significantly influence the success rate. Although our study did highlight that the unsuccessful group had a higher mean number of previous ectopic pregnancies, this did not show statistical significance. An interesting factor in our data was a higher success rate among the local population (67.2%) compared to the non local population (37.2%). This indeed is a variable not observed in several other studies but this could be attributed to a later presentation of the non local population to health services and poor compliance with follow ups. Upon monitoring the β-hCG levels throughout days four, seven, and 21, our study showed that both successful and unsuccessful groups had a complete resolution around the same time (Table 3). This may be explained by the fact that the unsuccessful group was referred shortly to surgical intervention after the failure of medical therapy, hence having a shorter resolution time. β-hCG levels that achieved a successful outcome on days one to seven and days four to seven indicated that there was a very low chance of random results that may weaken our study as the curve is far away from the line of random classification in all Figures 1-4.

It is also worth mentioning, that the successful treatment group had a 50% fall in β-hCG levels from day one to seven compared to only 10% in the unsuccessful group. This indicated that most cases with successful treatment will show a remarkably significant decline during days one to seven. In comparison to our study, Skubisz, et al. reported a more than 20% fall of β-hCG during days 0 to four versus our reported 15% fall between days four to seven, given that their pre-treatment β-hCG level was <3000 IU/L [17]. According to Agostini et al., the cut-off level of a 20% drop in β-hCG levels between days one to four predicted the success rate with 97% sensitivity [18]. For instance, according to Ozyuncu et al. three cervical and four caesarean scar ectopic pregnancies were also successfully treated with methotrexate and no further surgical intervention was required [19]. Finally, methotrexate is a cost-effective therapeutic alternative to surgical intervention and a single dose of methotrexate can have a success rate of up to 84% [20]. It is therefore, a favourable option when well defined criteria are met which includes an asymptomatic patient and a low pre treatment β-hCG level as the most important criteria.

The difference in the success rate between the local and non-local population represents potential selection bias but as a factor in itself should not affect our findings as local population did have different mixed ethnicity background and the obvious difference in the success rate is circumstantial due to access to medical service issue. Moreover, the retrospective nature of the study was a prominent limitation during the data collection phase due to the frequent absence of data on potential confounding factor. Additionally, this type of study can be prone to recall or misclassification bias. The small sample size is another limitation of this study as it may make it difficult to determine the accuracy of the study findings.

## Conclusions

In conclusion, the use of methotrexate for the medical treatment of ectopic pregnancy is a well-known common practice and can be considered the first line treatment option for ectopic pregnancies if selection criteria are met as it becomes a safe and effective choice for most women. Following the pattern of several studies, our study found that low initial β-hCG levels can be used to predict successful methotrexate treatment of ectopic pregnancy. In this cohort of patients, the cut-off level of initial β-hCG for successful treatment was 2141 IU/L. Eventually, these factors can further improve the accuracy of predicting a successful medical treatment, thereby preventing potential surgical interventions.

## References

[1] Mann LM, Kreisel K, Llata E, Hong J, Torrone EA (2020). Trends in ectopic pregnancy diagnoses in United States Emergency Departments, 2006-2013. Matern Child Health J.

[2] (2021). Current trends ectopic pregnancy -- United States, 1990-1992. https://www.cdc.gov/mmwr/preview/mmwrhtml/00035709.htm.

[3] Ankum WM, Mol BW, Van der Veen F, Bossuyt PM (1996). Risk factors for ectopic pregnancy: a meta-analysis. Fertility and sterility.

[4] (2018). ACOG Practice Bulletin No. 193: Tubal Ectopic Pregnancy. Obstet Gynecol.

[5] Alur-Gupta S, Cooney LG, Senapati S, Sammel MD, Barnhart KT (2022). Two-dose versus single-dose methotrexate for treatment of ectopic pregnancy: a meta-analysis. Am J Obstet Gynecol.

[6] Zhang J, Zhang Y, Gan L, Liu XY, Du SP (2020). Predictors and clinical features of methotrexate (MTX) therapy for ectopic pregnancy. BMC Pregnancy Childbirth.

[7] Forhad QE, Begum M, Alam IP, Alam MS (2013). Safety and efficacy of methotrexate in unruptured ectopic pregnancy. Bangladesh J Obstet Gynecol.

[8] Morlock RJ, Lafata JE, Eisenstein D (2000). Cost-effectiveness of single-dose methotrexate compared with laparoscopic treatment of ectopic pregnancy. Obstetrics & Gynecology.

[9] Bachman EA, Barnhart K (2012). Medical management of ectopic pregnancy: a comparison of regimens. Clin Obstet Gynecol.

[10] Levin G, Dior U, Shushan A, Gilad R, Benshushan A, Rottenstreich A (2019). Early prediction of the success of methotrexate treatment success by 24-hour pretreatment increment in HCG and day 1-4 change in HCG. Reprod Biomed Online.

[11] Panelli DM, Phillips CH, Brady PC (2015). Incidence, diagnosis and management of tubal and nontubal ectopic pregnancies: a review. Fertil Res Pract.

[12] Lipscomb GH, McCord ML, Stovall TG, Huff G, Portera SG, Ling FW (1999). Predictors of success of methotrexate treatment in women with tubal ectopic pregnancies. N Engl J Med.

[13] Murray H, Baakdah H, Bardell T, Tulandi T (2005). Diagnosis and treatment of ectopic pregnancy. CMAJ.

[14] Barnhart KT, Gosman G, Ashby R, Sammel M (2003). The medical management of ectopic pregnancy: a meta-analysis comparing “single dose” and “multidose” regimens. Obstetrics & Gynecology.

[15] Richardson A (2012). Medical management of ectopic pregnancy: a 10-year case series. Hum Fertil (Camb).

[16] Natale A, Busacca M, Candiani M, Gruft L, Izzo S, Felicetta I, Vignali M (2002). Human chorionic gonadotropin patterns after a single dose of methotrexate for ectopic pregnancy. Eur J Obstet Gynecol Reprod Biol.

[17] Skubisz M, Dutton P, Duncan WC, Horne AW, Tong S (2013). Using a decline in serum hCG between days 0-4 to predict ectopic pregnancy treatment success after single-dose methotrexate: a retrospective cohort study. BMC Pregnancy Childbirth.

[18] Agostini A, Blanc K, Ronda I, Romain F, Capelle M, Blanc B (2007). Prognostic value of human chorionic gonadotropin changes after methotrexate injection for ectopic pregnancy. Fertil Steril.

[19] Ozyuncu O, Tanacan A, Duru SA, Beksac MS (2018). Methotrexate therapy for ectopic pregnancies: a tertiary center experience. Rev Bras Ginecol Obstet.

[20] Stovall TG, Ling FW (1993). Single-dose methotrexate: an expanded clinical trial. Am J Obstet Gynecol.

